# SpillOver stimulation: A novel hypertrophy model using co-contraction of the plantar-flexors to load the tibial anterior muscle in rats

**DOI:** 10.1371/journal.pone.0207886

**Published:** 2018-11-20

**Authors:** Martin Schmoll, Ewald Unger, Hazel Sutherland, Michael Haller, Manfred Bijak, Hermann Lanmüller, Jonathan Charles Jarvis

**Affiliations:** 1 Center for Medical Physics and Biomedical Engineering, Medical University of Vienna, Vienna, Austria; 2 School of Sport and Exercise Sciences, Liverpool John Moores University, Liverpool, United Kingdom; University of Tennessee Health Science Center College of Graduate Health Sciences, UNITED STATES

## Abstract

The influence of loading on muscular hypertrophy has previously been studied in rodents by removal of synergistic muscles or various weight-lifting regimes. We present a novel model, evoking hypertrophy in the ratʹs tibialis anterior (TA) muscle by means of an implanted single channel electrical nerve stimulator. The amount of load experienced by the TA was measured in acute experiments in anaesthetized rats with contractions over a range of stimulation frequency and amplitude. A novel electrode configuration allowed us to elicit concentric, isometric and eccentric contractions within the same setup. This was achieved by ‘SpillOver’ stimulation in which we adjusted the amount of co-activation of the stronger antagonistic plantarflexors by increasing the stimulus above the level that caused full recruitment of the dorsiflexor muscles. The effect of loading on hypertrophy of the TA was tested in 3–4 week stimulation experiments in two groups of freely-moving rats, with a protocol that resembles typical resistance-training in humans. One group performed concentric contractions with no antagonistic co-contraction (unloaded, UNL, n = 5). In the other group the TA was loaded by simultaneous co-contraction of the antagonistically acting plantarflexors (SpillOver, n = 5). The wet mass of the stimulated TA increased in both groups; by 5.4 ± 5.5% for the UNL-group and 13.9 ± 2.9% for the SpillOver-group, with significantly greater increase in the SpillOver-group (p<0.05). Our results correlate well with values reported in literature, demonstrating that SpillOver-stimulation is a suitable model in which to study muscular hypertrophy. Even higher gains in muscle-mass may be possible by optimizing and adjusting the stimulation parameters according to the principles of progressive resistance training.

## Introduction

Living organisms have a remarkable ability to adapt to a wide range of environmental changes. This ability can be used to trigger desired responses. In healthy subjects a coordinated increase in the daily amount of physical activity can induce changes within the cardiovascular system [1], increases in muscle strength and endurance [2], altered body-composition [3], and cognitive and psychological functioning [4] leading to an improved fitness and well-being. In mammalian skeletal muscle, goal-oriented progressive resistance training can induce adaptations of the cell-biology, physiology and neuromuscular control of the muscle [5] in order to efficiently fulfil the demands of a particular locomotion task. The American College of Sports Medicine (ACSM), as an example, provides training guidelines for muscular characteristics such as strength, hypertrophy (muscle mass), power and endurance [2]. However, there is still a need for a more explicit understanding of the cell biology regarding the relationship between activity, loading and cellular response.

In an increasingly sedentary society, maintenance of muscular mass and strength is a crucial factor to ensure quality of life. Protein-synthesis and protein-degradation are influenced by the daily activity of the muscle [6]. When aiming for increases in mass of a particular muscle (hypertrophy), high tension, stretch and muscle damage [6] are well known anabolic signals leading to increased protein-synthesis. The ACSMʹs recommendation for hypertrophy is to perform exercises at an intensity of 75–85% of the one repetition maximum, with 8–12 repetitions, in 1–3 sets per session with a rest of 1–2 min between each set. Such training is recommended to be repeated 2–3 times a week. While such recommendations are mainly based on studies conducted on healthy young adults, the underlying principles for the ageing population or subjects with musculoskeletal disorders are less clear and require further investigation. Sarcopenia, an age-related loss of skeletal muscle mass, is identified being a main cause of functional decline and loss of independence in older adults [7]. We need to understand the cell biology of muscular hypertrophy in order to provide rational training strategies [8] to counteract muscular atrophy.

Aiming for a deeper understanding of mechanisms involved in hypertrophy, various animal models were used in the past in which the average loading per day of particular muscles was increased. Some models investigated the effect of compensatory hypertrophy by surgical removal [9–11] or denervation [12] of synergistic muscles, producing a constant overload. This rather invasive approach demonstrates the remarkable ability of skeletal muscle to react to a chronic increase in loading by rapidly increasing its mass, but is not directly applicable to resistance training for human health.

Other groups used weight-lifting strategies to establish a training-protocol in intact rats intending to mimic human resistance training to study the influence of increased muscular effort. The animals were encouraged to execute weighted squat-like exercises [13–16] or to climb ladders with weights attached to their tail [17–19]. Prior to the actual training, the animals were prepared to execute the exercise properly. To motivate the animals to comply with the protocol, methods like food reward after a period of reduced food supply [14,16] or the application of electric shocks to initiate movement [13,15] were applied. It remains challenging to ensure complete and reproducible execution of the demanded task. Further it must be considered that food restriction as a motivating factor could have influenced the outcome of the hypertrophic response [20].

Alternatively electrical nerve stimulation can be used to elicit contractions. Although the recruitment order of muscle fibers differs from the muscles natural activation [21], it is a powerful tool in research and therapy. Electrical motor nerve stimulation to activate muscles has been used in hypertrophy studies to train anaesthetized animals [22–24], to ensure a reliable and comparable execution of the intended training-protocol. Unfortunately the proposed techniques still require a lot of manual interaction and multiple periods of anesthesia to conduct the experiment which leads to increased stress for the animal and may affect the hypertrophic response.

Advances in modern micro-electronics have allowed progressive miniaturization of implantable pulse generators (IPG) while simultaneously increasing their functionality and lifetime. Such implantable devices minimize the amount of disturbance to the animal model and offer a great variety of possible applications. While early devices produced a simple constant series of stimuli at a fixed frequency [25,26], modern devices can deliver complex daily stimulation patterns autonomously [27].

Our main goal was to investigate and validate a novel hypertrophy model which uses adjustable antagonistic co-contraction by means of implantable electrical stimulators, to produce loaded (resisted) contractions of the tibialis anterior (TA) muscle in freely moving rats. While certain guidelines for human resistance training are already well established, there is still no general agreement on optimal parameters when using functional electrical stimulation for training. In order to design an appropriate training protocol it is essential to know the type of loading achievable with this technique. The tensile force transmitted by the TA-tendon, which is equivalent to the force experienced by the TA-muscle, was assessed in acute experiments using a novel miniature in-line load-cell. The forces measured during unloaded (UNL) and isometric (ISO) contractions with activation via the common peroneal nerve (CPN stimulation) were compared with the forces that were achieved when simultaneously activating the fully recruited CPN and some of the axons in the tibial nerve (SpillOver stimulation). The tibial nerve supplies the plantarflexor muscles that act antagonistically across the ankle joint and thus resist the contraction of the TA.

A high-intensity stimulation pattern which was based on the outcome of these force-measurements was used in a second series of experiments, to train the TA-muscle for a period of several weeks. We hypothesized that the higher tension generated during SpillOver contractions would cause greater gains in hypertrophy than in relatively unloaded contractions.

## Material and methods

### Animals

All experiments were conducted under the permission of the Animals (Scientific Procedures) Act 1986 and approved by the British Home Office (PPL 40/3280). This study used 15 male adult Wistar rats which were bred within our local animal unit. All animals were group-housed maintaining an alternating 12 h light 12 h dark cycle. Food and water was supplied ad libitum and all rats underwent daily health checks by our local staff. The 15 rats were divided into three groups of 5 animals each.

One group (UNTRAINED, 83 ± 11 days, body mass = 316 ± 15 g) served as control and was used to perform physiological measurements under anesthesia during acute experiments. The animals of the other two groups underwent a high-intensity-resistance training by means of electrical nerve stimulation for 3–4 weeks intended to induce hypertrophy of the TA muscle.

The first training group (UNL, n = 5, age: 47± 2 days, body mass = 310 ± 8 g) received isolated stimulation of the common peroneal nerve (two electrodes under CPN), which resulted in relatively unloaded shortening contractions of the TA muscle.

In the other group of trained animals (SpillOver, n = 5, age: 198 ± 52 days, body mass = 450 ± 21 g) stimulation was achieved via a different electrode placement. The cathode was placed underneath the CPN while the anode was positioned underneath the tibial nerve. SpillOver stimulation takes advantage of the different stimulation thresholds for anodic and cathodic stimulation. The lower stimulation threshold at the cathode results in an initial recruitment of all the axons of the CPN followed by additional depolarization of some motorneurones within the tibial nerve at higher stimulation amplitudes. We set an amplitude that provided enough activation of the plantarflexors to resist the action of the dorsiflexors so that the ankle angle did not decrease. The forces produced by the much stronger plantarflexor muscles were transmitted via the ankle joint and caused additional loading in auxotonic contractions of the TA muscle.

The hind limb muscles TA, extensor digitorum longus (EDL), plantaris (PLA), soleus (SOL) and gastrocnemius (GAST) from the left and right side were harvested from all animals and weighed immediately after termination of the experiment.

### Acute physiological measurements

#### Surgical procedure

All surgical procedures were performed with the animal anaesthetized using a gaseous mixture of isoflurane and O_2_. An initial concentration of 4% was used for induction and was adjusted individually to levels of 1–2% to maintain adequate anesthesia. Buprenorphine (Temgesic, Indivior, Slough, UK) at a dose of 0.05 mg kg^-1^ body mass, was administered for analgesia.

Three loop electrodes, formed from PVC-insulated stainless steel wires (Cooner Sales Company, Chatsworth, California, U.S.A.), were positioned as shown in 1-A. Two electrodes were placed under the common peroneal nerve with the more distal electrode as cathode. A third electrode, placed under the tibial nerve, was used simultaneously to activate parts of the plantar-flexor musculature (SpillOver stimulation). The cathode was the same in both electrode configurations. The incision was closed in layers after electrode placement to maintain the nerve in its natural environment.

**Fig 1:**
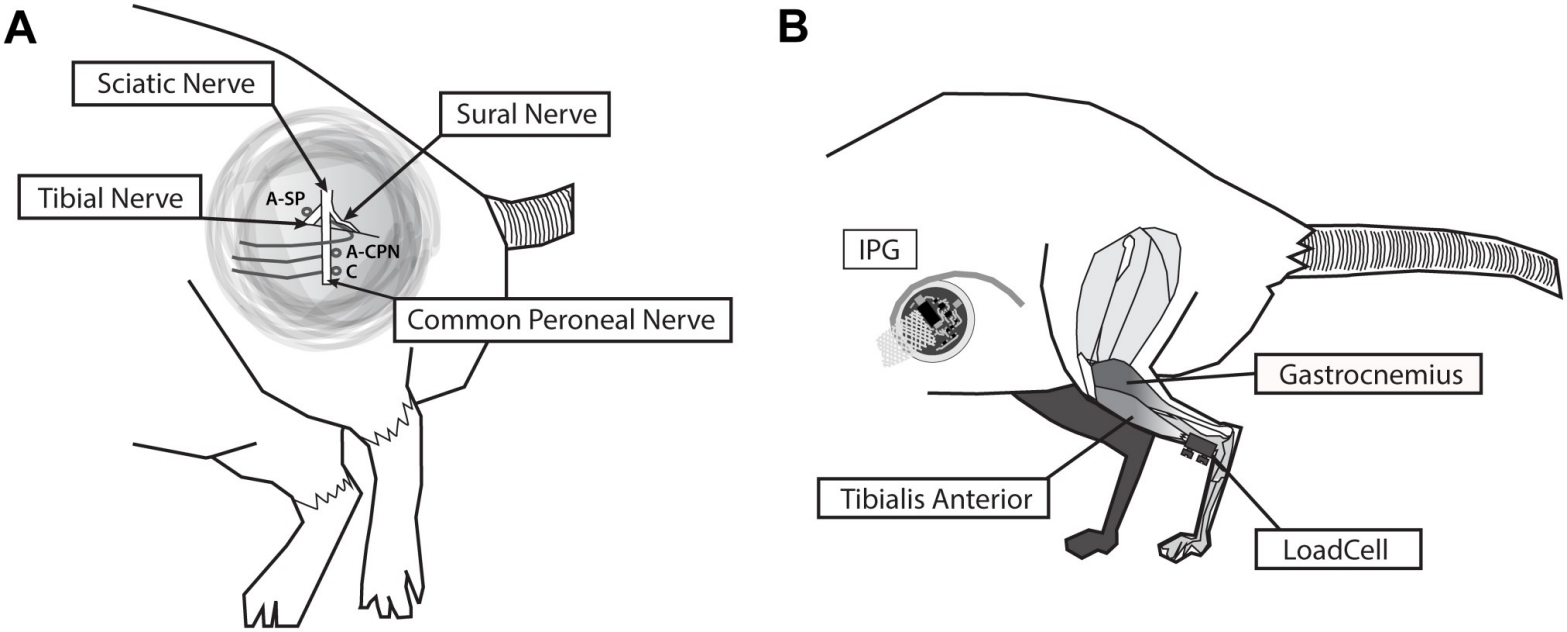
Schematic of the experimental setup. A) Electrode placement for the isolated stimulation of the common peroneal nerve [Anode: A-CPN, Cathode: C] and SpillOver stimulation of common peroneal and tibial nerve [Anode: A-SP, Cathode: C]. B) Position of the in-line loadcell used to measure tensile forces acting on the tibialis anterior muscle. Illustration to approximate scale of the implanted pulse generator (IPG) used for electrical stimulation during the high-intensity-resistance training.

To measure forces transmitted through the TA tendon, a custom-built load cell was attached as described previously [28]. Due to its position (see Fig 1B) in-series with the natural pathway of force transmission, it is possible to measure forces that are generated by the TA itself, as well as additional forces transmitted via the ankle joint and TA tendon.

#### Stimulation equipment

Passively charge-balanced, pseudo-monophasic, rectangular pulses, were delivered from a pulse generator (MiniVStim Type 12B, Competence Team for Implanted Devices, Center for Medical Physics and Biomedical Engineering, Medical University Vienna, Austria). All stimulation patterns were implemented using the MiniVStim-App installed on a standard Android driven tablet computer (Xperia Tablet Z, Sony Corporation, Tokyo, Japan). The tablet computer maintained an active Bluetooth connection to a MiniVStim programming device which served as bridge to communicate with the pulse generator via an additional radio frequency (RF) link.

#### Data recording

A PowerLab 16/35 (ADInstruments Inc., Colorado Springs, USA) device was used to supply the driving voltage for the load cell and to measure the force signal at 10 kS/s. Stimulation voltage was also recorded at a sample rate of 100 kS/s. ADInstruments LabChart 7 Pro (ADInstruments Inc., Colorado Springs, USA) installed on a standard personal computer (MSI GS60 2PE Ghost Pro, Micro-Star International, Zhonghe District, Taiwan) was used to record, store, pre-process and export the retrieved data.

#### Stimulation protocol

Recruitment curves: Single charge-balanced monophasic pulses with a phase-width (PhW) of 258 μs were delivered every 3 s while the stimulation amplitude was increased every other pulse by 0.1 mA, over the range of amplitude levels from 0.1 to 2.0 mA. The mean force response elicited by two consecutive pulses with the same amplitude was used to create recruitment curves in three experimental conditions. In a first step, measurements were performed with the foot free to move, allowing for unloaded concentric contractions of the TA muscle. That is, the muscle acted only against the mass of the foot, which was free to dorsiflex in air. Isometric contractions were achieved by restraining the animalʹs foot by hand with the ankle joint at approximately 90 °. Finally, contractions using SpillOver stimulation were measured allowing the foot to move freely again, but with the dorsiflexion resisted internally by some active plantar flexor activity.

Force frequency curves: The relation between developed force and stimulation frequency was assessed with 300 ms bursts at frequencies of 10, 20, 40, 60, 80 and 100 Hz. The time between bursts was set to 30 s, to minimize fatigue and allow for full recovery after contraction.

Forces were recorded for UNL and ISO contractions using a stimulation amplitude of 2 mA (supra maximal for the CPN). For contractions using SpillOver stimulation, the amplitude was varied from 0.1 mA to 2 mA in 0.1 mA steps for all frequencies.

Single frequency-ramped bursts as described earlier [29] were also tested as an alternative time-saving method to estimate the force frequency relationship. Bursts with a supra-maximal stimulation amplitude of 2 mA were applied in the UNL and ISO regime, while SpillOver stimulation used amplitudes of 0.5, 1.0, 1.5 and 2.0 mA to provide a range of plantarflexion force.

Conditioning pattern: Based on the ACSM recommendations [2] for human resistance training we designed a stimulation pattern intended to induce hypertrophy in the TA muscle using sets of brief tetanic contractions in a single ~13 minute session each day. Five sets of ten repetitions consisting of 2 s stimulation (F = 100 Hz, PhW = 258 μs, I = 1 mA) and 2 s rest were delivered with a break of 2.5 min between the sets.

We measured acutely the change in force generated in response to the conditioning pattern delivered to the SpillOver electrode configuration. This pattern was used in the subsequent hypertrophy training experiments.

#### Data analysis

LabChart was used to mark the beginning of each twitch and burst. This annotated data was exported for further processing with Matlab R2010a (MathWorks, Natick, Massachusetts, United States). A semi-automatic script was used to analyze the recorded data. To evaluate the active force developed by the muscle, a manually selected baseline (passive force) was subtracted from the force-signal. The peak force (F_peak_) was determined for every single response. Results were stored and further processed using Microsoft Excel (Microsoft Corporation, Washington, United States). One-way ANOVA with a selected significance level of α = 0.05 was used to test statistical differences.

Alignment recruitment curves: The lowest stimulation amplitude that was able to elicit a response with F_peak_ > 0.1 N was considered the threshold amplitude. The threshold is influenced by the position of the stimulation electrodes and can be slightly different for every animal, which made it necessary to align the recruitment curves before calculating average peak-force values for a particular amplitude level. Amplitude is thus analyzed and presented as amplitude-above-threshold.

### Chronic resistance training

#### Surgical procedure

For the two training groups, silicone encapsulated RF controlled IPGs (MiniVStim 12B, Competence Team for Implanted Devices, Center for Medical Physics and Biomedical Engineering, Medical University Vienna, Austria) were used to deliver electrical stimulation over a period of 3–4 weeks. The devices were implanted into the abdominal cavity accessed by a lateral incision through skin and peritoneum, directly below the rib cage on the left side.

A polyester mesh attached to the implant was incorporated into the suture line closing the peritoneum, securing the position of the device. Two PVC-insulated stainless-steel electrode leads (Cooner Sales Company, Chatsworth, California, U.S.A.), with terminal conductive loops, were fed through the peritoneal incision and tunneled under the skin to their final destination. A second lateral incision through skin and biceps femoris muscle revealed access to the CPN. In animals of the UNL stimulated group both electrodes were placed under the CPN, with the more distal electrode being the cathode.

For the SpillOver stimulated group the incision was carefully extended further proximal, along the CPN, up to the level where the sciatic nerve branches into tibial, common peroneal and sural nerves. The anode was placed beneath the tibial nerve while the cathode again was situated under the common peroneal nerve. All incisions were closed in layers and the animal was given at least 3 days rest to recover.

An additional dose of Buprenorphine was available directly after surgery if the animal showed any signs of discomfort and on the first post-operative day. Wound healing status and a general health check was performed every day by the local staff of the animal unit.

After the training period the animals were sacrificed by a slowly rising concentration of carbon dioxide CO_2_, followed by cervical dislocation. TA, EDL, PLA, SOL and GAST muscles from the left and right side were harvested and weighed immediately.

A transverse block approximately 5 mm long was cut from the central part of each muscle and mounted on a labelled cork disk using cryostatic mounting media (Tissue-Tek^®^ O.C.T. Compound, Sakura Finetek USA Inc, Torrance, CA, USA). The remaining muscle parts were separately wrapped in tinfoil, labelled and frozen in liquid nitrogen. The cork mounted muscle samples were frozen in melting isopentane above liquid nitrogen to prevent freezing damage before putting them into liquid nitrogen. All muscle samples were stored at -80 °C till further processing.

#### Training protocol

Both the UNL and the SpillOver group, were trained every 24 h with the conditioning pattern (5 sets with 10 contractions each 2 s stimulation followed by 2 s rest; rest between sets 2.5 min) described in the physiological measurements section for 21.0 ± 0.0 days and 25.8 ± 2.8 days, respectively. The stimulation was conducted in the left hind limb while the right hind limb provided unstimulated control muscle tissue. Prior to the daily training, an accommodation pattern (F = 4 Hz, PhW = 258 μs, I = 1 mA) was applied for 10 s. It was not necessary to administer anesthesia during the period of training as the electrical stimulation was tolerated very well by all animals (regular observations during the 4 weeks of daily training revealed no adverse effects).

#### Histological analysis

Histologic sections with a thickness of 5 μm were taken from the left and right TA and EDL muscles using a cryostat (OTF5000, Bright Instruments Ltd, Luton, United Kingdom) and collected on subbed microscope slides. The sections were labelled with a fluorescein wheat germ agglutinin (FL-1021, Vector Laboratories Ltd, Peterborough, United Kingdom) diluted in phosphate-buffered saline (PBS) at a concentration of 25 μg/ml. After an incubation time of 60 min in the dark, the solution was rinsed off carefully with PBS. To preserve fluorescence, the sections were covered with Vectashield^®^ (H-1200, Vector Laboratories Ltd, Peterborough, United Kingdom), a mounting medium with DAPI (4’, 6-diamindino-2-phenylindole). A cover slip was placed on top of the sections and was sealed with nail varnish to keep the mounting medium on top of the section during handling.

**Fig 2:**
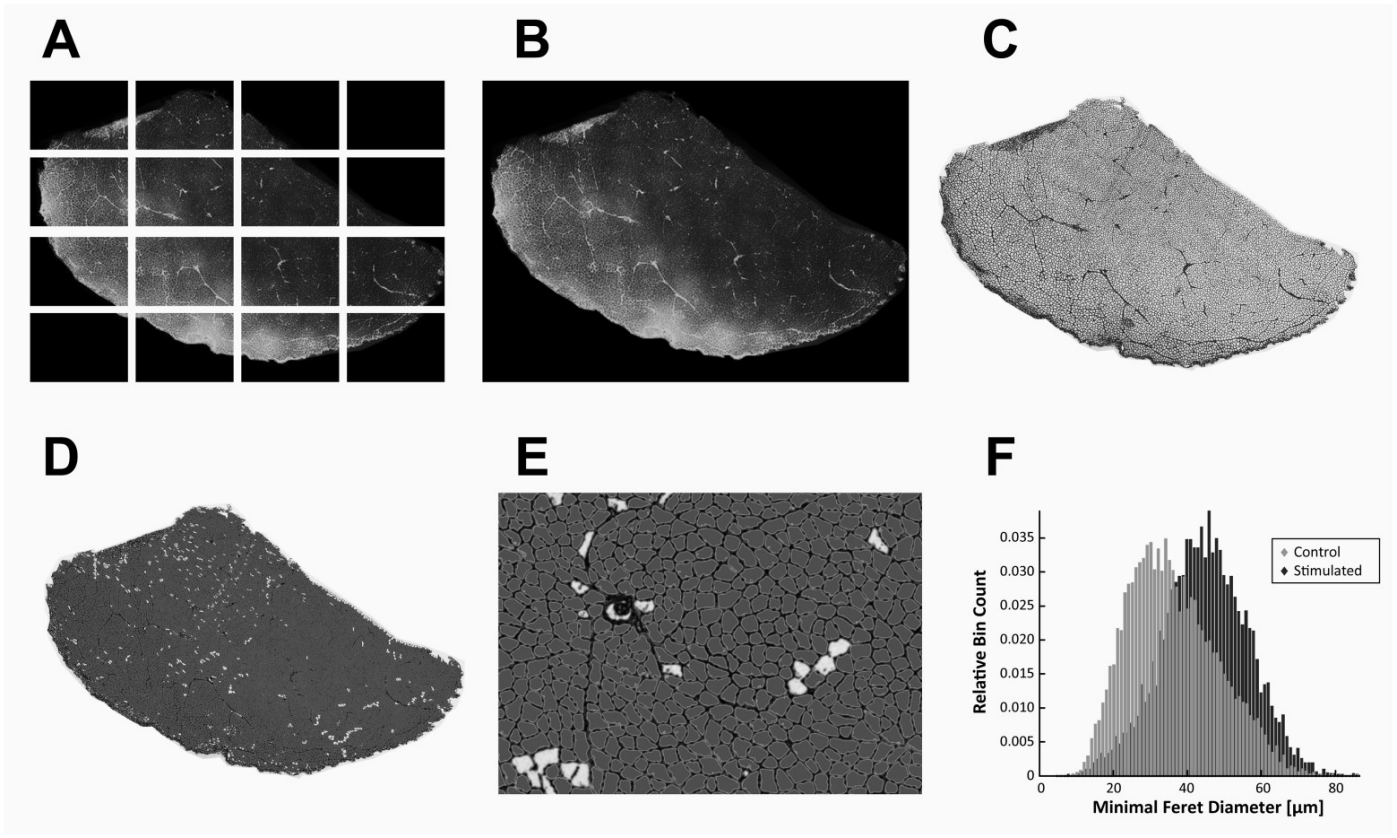
Image processing and automated cell analysis. Illustration of the individual steps from image acquisition to cell analysis performed for each muscle section. A, B) Single fluorescent microscopic images were merged to obtain a full-scale image of the entire muscle section at an appropriate magnification to see individual muscle fiber outlines. C) The contrast of the overall images was enhanced by edge detection. D, E) A commercial histogram based segmentation algorithm automatically performed measurements of cross sectional area (CSA) and minimal Feret’s diameter for each detected object. Objects not fulfilling certain criteria (area >250 μm^2^, perimeter < 430 μm, roundness < 2) were considered artefacts and excluded from further analysis. F) shows distributions of Minimum Feret’s diameter from a typical control and a stimulated muscle.

The most complete section on each slide was selected to be digitized. Fig 2 gives a general overview of the digitization and image analysis procedure. A fluorescence wide-field transmission microscope (DM6000, Leica, Wetzlar, Germany) excited the sections at a wavelength of 500 nm and recorded the emitted light with a monochrome digital camera (DFC365FX, Leica, Wetzlar, Germany) to take a series of photographs that covered the whole section. The single pictures were combined using the photo-merge feature of Photoshop (Version CS6, Adobe Systems, San Jose, California, United States) to obtain a complete image of each cross-section at full resolution. Selecting the “Reposition” option of this feature ensured that the individual images were not resized or distorted.

The merged images were further processed with Photoshop to prepare them for automatic segmentation. Edge detection was applied on the images to compensate for uneven illumination and to increase the contrast. To close remaining small gaps along the cell outlines, the images were slightly blurred using a 2 px Gauss filter.

The automatic segmentation and cell-based measurements were performed using Image Pro Analyser (Version 7.0, Media Cybernetics Inc., Maryland, USA). Histogram based segmentation with a brightness-threshold of 160 was used to differentiate background and cell-boundaries. The software automatically detected connected objects using a 4-connect growing algorithm. Objects at all borders were excluded from the analysis. Remaining objects were filtered according to their area (> 250 μm^2^), perimeter (< 430 μm) and roundness (< 2) to exclude structures other than muscle cells. All parameters were obtained empirically and were applied consistently to all the different images.

For each cell, fiber cross-sectional area (CSA) and minimal Feret’s diameter (MFD) were measured and stored for further analysis. To compensate for obliquely cut fibers within the muscle, the MFD was used to calculate an estimated fiber cross-sectional area (eCSA) assuming a circular shape of the muscle fiber cross-section. Matlab was used to analyze the CSA, MFD and eCSA to obtain a histogram extracting the statistical descriptors mean, first quartile (Q1), second quartile (= median, Q2), third quartile (Q3) and inter-quartile-distance (Q3-Q1) for the trained and the control side. To compare the individual animals, the differences between trained (left) and control (right) side were calculated and normalized to the control side. One-way ANOVA with a selected significance level of α = 0.05 was used to test statistical differences between CPN and SpillOver stimulation.

## Results

### Acute physiological measurements

#### Recruitment curves

**Fig 3:**
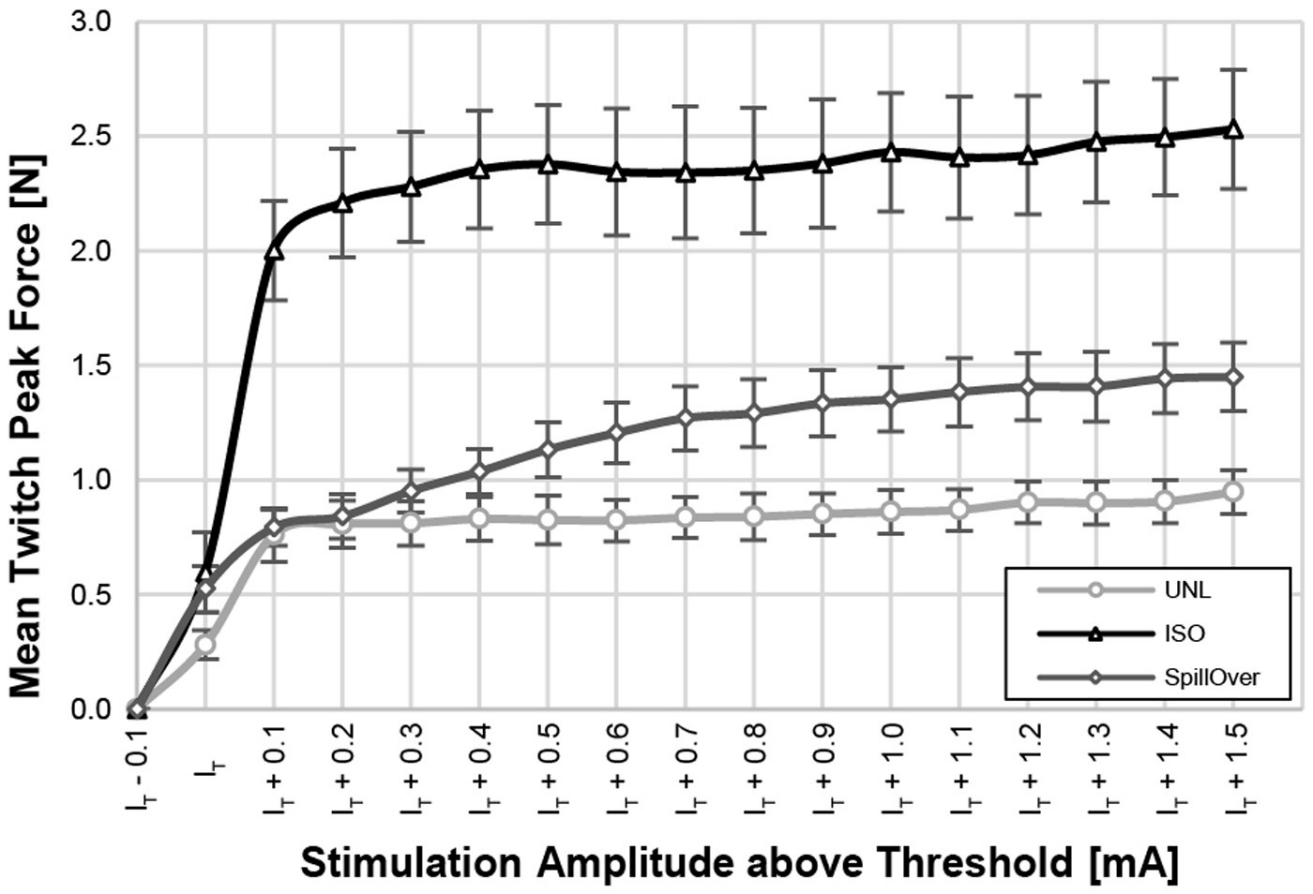
Averaged recruitment curves. Recruitment curves were obtained using single pulses to elicit unloaded concentric (light grey), isometric (black) and SpillOver (dark grey) twitches. Individual recruitment curves were aligned on the x-axis according to their threshold amplitude (I_T_), before calculating the mean peak-force of each amplitude step above threshold. Data is presented as mean twitch peak force ± SEM. The average I_T_ and SEM was 0.46 ± 0.05 mA for unloaded, 0.44 ± 0.04 mA for isometric and 0.34 ± 0.02 mA for SpillOver contractions. There were no significant differences between the threshold amplitudes.

Recruitment curves were recorded for unloaded concentric, isometric contractions and contractions elicited by SpillOver stimulation. Threshold-aligned recruitment-curves are illustrated in Fig 3 for the different loading regimes. The mean threshold amplitude and SEM of UNL, ISO and SpillOver were 0.46 ± 0.05 mA, 0.44 ± 0.04 mA and 0.34 ± 0.02 mA, respectively.

An overview of the maximal peak twitch forces measured for each animal can be found as supporting material (S1 Table). The mean maximum twitch forces and SEM were 0.96 ± 0.09 N for UNL, 2.56 ± 0.27 N for ISO and 1.45 ± 0.15 N for SpillOver twitches. Peak forces were significantly higher for both ISO (+167%, p < 0.05) and SpillOver (+51%, p < 0.05) contractions compared to the UNL case.

#### Force-frequency curves

**Fig 4:**
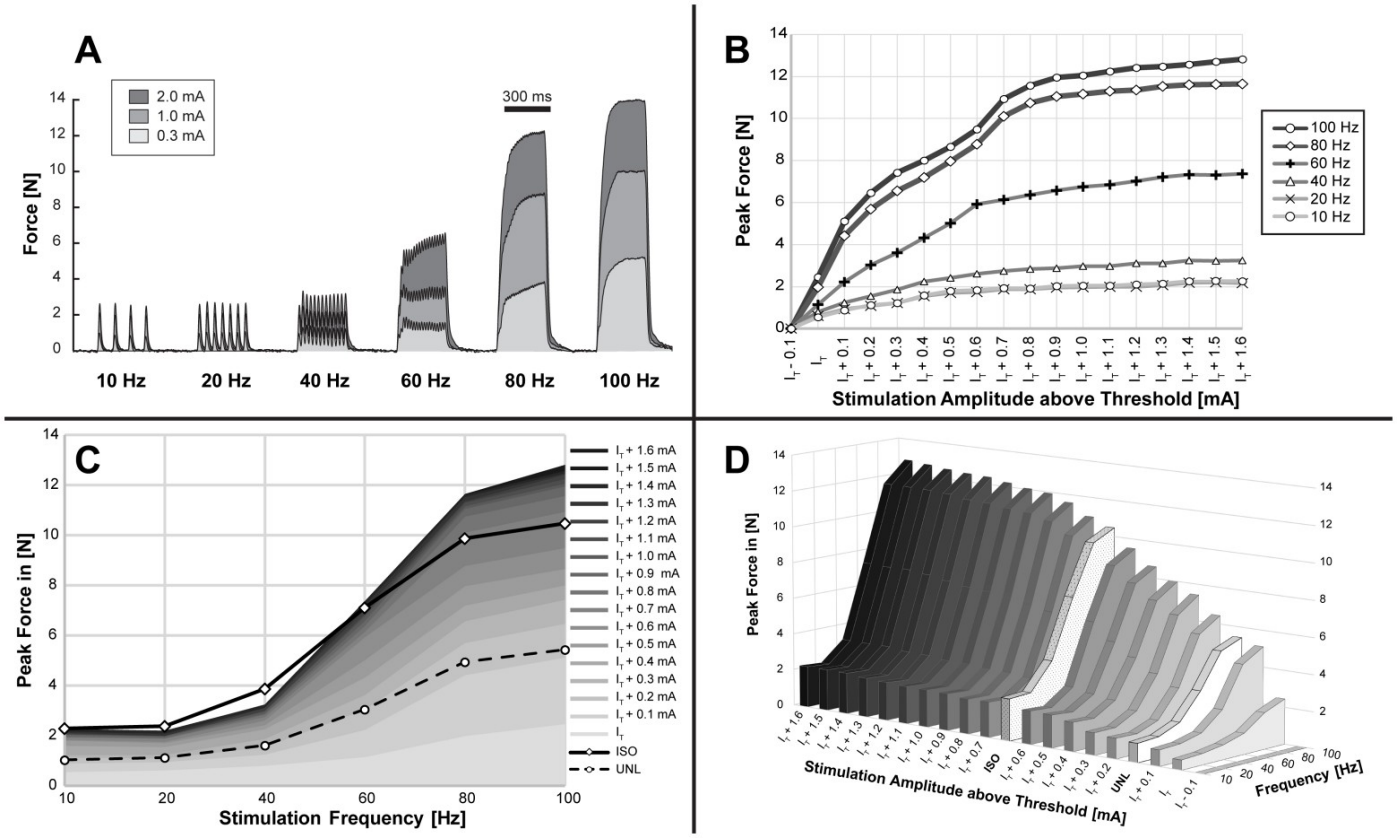
Force frequency relationship. The force frequency relationship of the tibialis anterior muscle was measured acutely in 5 anaesthetized rats (UNTRAINED group) for different loading regimes. Activation of the muscle was achieved by applying short [300 ms] electrical stimulation bursts of 10, 20, 40, 50, 80, and 100 Hz and amplitudes between 0.1 and 2.0 mA. A) Representative measurements of the tensile forces generated during SpillOver stimulation. B) Averaged recruitment curves obtained for different frequencies using SpillOver stimulation. Individual recruitment curves were aligned according to their threshold amplitude (I_T_), before calculating the mean developed force for each amplitude step above threshold. Data is presented as mean peak force and SEM. C&D) Averaged force frequency curves for supra-maximal stimulation of the common peroneal nerve at unloaded concentric (UNL) and isometric (ISO) contractions, along with SpillOver contractions at different amplitudes. Data is presented as mean peak force and SEM for UNL and ISO contractions, SpillOver contractions are only illustrated as mean peak force to provide a clear comparison.

Standard approach: Short (300 ms) supra-maximal (2 mA) bursts of different frequencies (10, 20, 40, 60, 80 and 100 Hz) were used to evaluate the force-frequency relationship of the TA muscle during UNL and ISO contractions. Additional measurements were performed using SpillOver stimulation at different amplitudes. An example is shown in Fig 4A. Increasing the amplitude from 0.1–2.0 mA in 0.1 mA steps resulted in recruitment curves for every frequency as illustrated in Fig 4B. The same data is presented as force-frequency curves Fig 4C and 4D.

With SpillOver stimulation it was possible to control the amount of loading of the TA muscle by adjusting the stimulation frequency and amplitude. Measured forces ranged from values below unloaded concentric contractions, up to forces greater than the isometric case.

Individual measurements of the maximal tetanic peak force, obtained with the different loading regimes are attached as supporting material (S2 Table). These forces were significantly higher for ISO (+93%, p < 0.05) and SpillOver (+139%, p < 0.05) than for UNL contractions.

**Fig 5:**
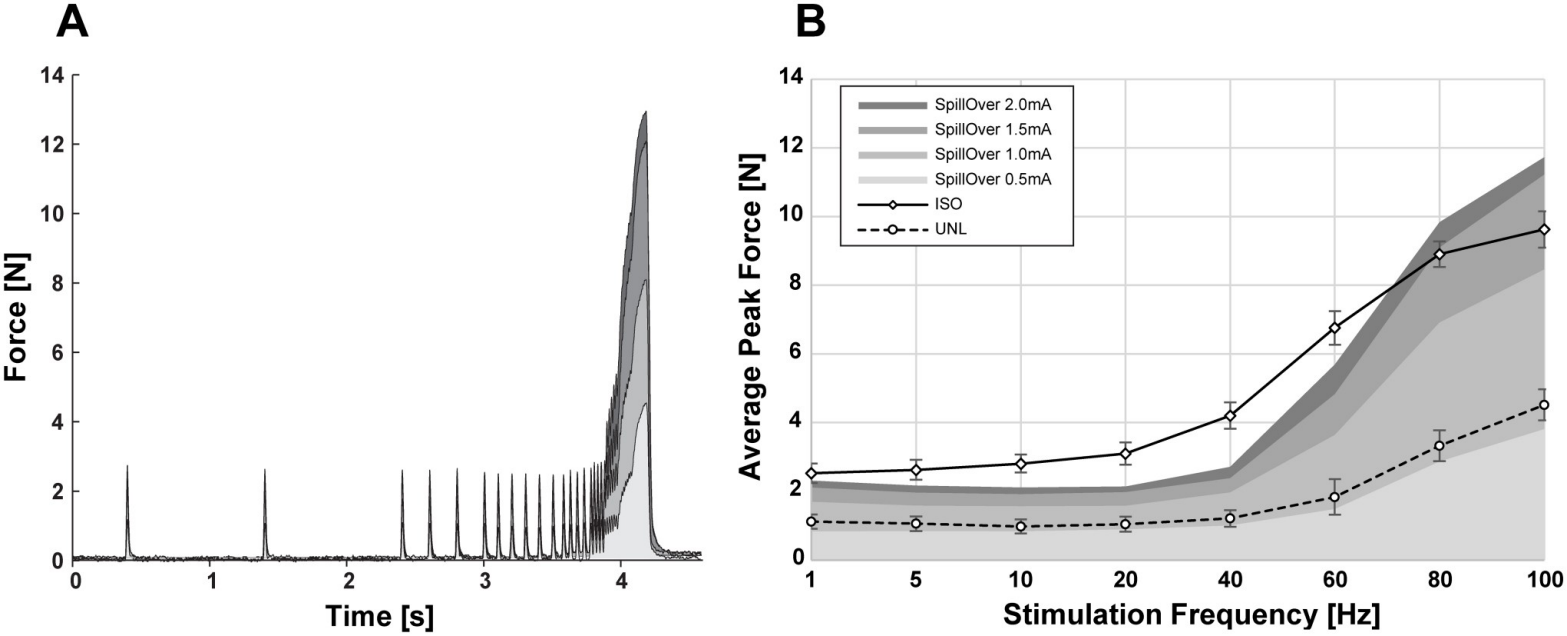
Frequency ramped bursts. A) Representative measurements of the tensile forces generated during application of a frequency ramped bursts lasting just less than 5s incorporating frequencies of 1, 5, 10, 20, 40, 60, 80 and 100 Hz at amplitudes of 0.5, 1.0, 1.5 and 2.0 mA using SpillOver stimulation. B) Averaged force frequency curves for supra-maximal stimulation of the common peroneal nerve at unloaded concentric (dashed black trace) and isometric (solid black trace) contractions, along with SpillOver contractions at different amplitudes. SpillOver stimulation at high frequency (above 70 Hz) was able to elicit forces in the tibialis anterior muscle higher than those produced during isometric contraction. Data is presented as mean peak force and SEM for UNL and ISO contractions, SpillOver contractions are only illustrated as mean peak force for better comparison.

Single-burst approach: Responses to frequency-ramped bursts were measured for UNL, ISO and SpillOver contractions (see Fig 5). Supramaximal (2 mA) elicited UNL and ISO contractions were compared with SpillOver contractions at 0.5, 1.0, 1.5 and 2.0 mA. Although the absolute peak force values are generally lower, because of the compromise necessary between time to develop force and fatigue within the burst, this technique gives a rapid estimate of the force-frequency relationship from a single compound contraction. The maximal tetanic peak force was significantly higher for ISO (+113%, p < 0.05) and SpillOver (+124%, p < 0.05) than for UNL contractions.

#### Conditioning pattern

**Fig 6:**
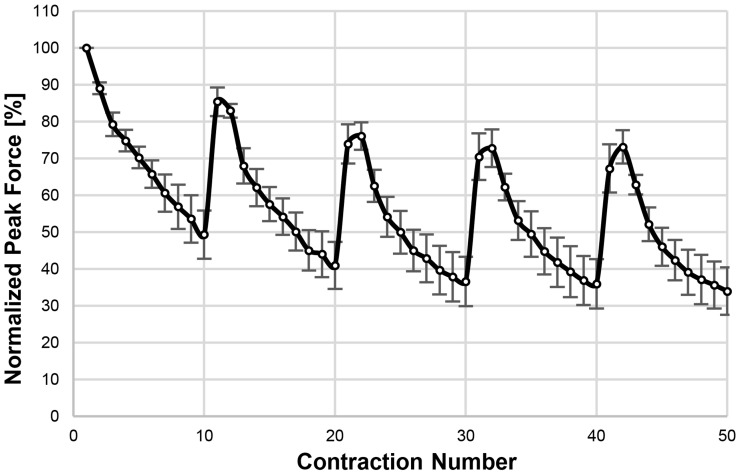
Conditioning pattern—Average peak forces. The average peak-forces of all animals in the untrained group (n = 5), normalized to the peak-force of the first contraction, w ere measured for a conditioning pattern delivered using SpillOver Stimulation at 1 mA and 100 Hz. The pattern consisted of 5 sets with 10 repetitions in each set (2 s contraction followed by 2 s rest) and 2.5 min rest between the sets. This pattern was used as the training pattern in the hypertrophy experiments. Data is presented as mean normalized peak force ± SEM. Note that x-axis is not time but contraction number in the training session, the figure does not depict the breaks between sets.

Average peak-forces, normalized to F_peak_ of the first contraction of the conditioning pattern were measured for each animal in the untrained group. The pattern produced a significant amount of muscular fatigue as illustrated in Fig 6. Actual values of force production capability (highest F_peak_ within a set) and fatigue (difference between highest and lowest F_peak_ within a set) can be found as supporting material S2 Table. The highest forces that the muscle was able to generate declined by in average about 25% from the first to the last set. The fatigue within each set was generally greater in the first two sets and remained almost constant for the consecutive sets.

We assume that this amount of loss of force may be compared with typical recommendations used for strength training in humans, that is, sets of contractions that cause failure to lift against the current load after about 8 repetitions. Different levels of fatigue might be achieved by adapting the parameters of the conditioning pattern.

### Chronic resistance training

#### Muscle weights

There was no significant difference in muscle wet mass between left and right side in the untrained group for any of the measured muscles (TA, EDL, GAST, PLA and SOL) (see Table 1).

**Table 1:** Muscle masses.

		TA		EDL		GAST		PLA		SOL	
	body weight	left	right	left	right	left	right	left	right	left	right
	g	mg	mg	mg	mg	mg	mg	mg	mg	mg	mg
Untrained	316 ± 15	623 ± 67	617 ± 105	140 ± 12	134 ± 14	1629 ± 168	1494 ± 157	317 ± 40	304 ± 27	133 ± 14	126 ± 15
UNL	310 ± 8	593 ± 46	562 ± 17	137 ± 5	132 ± 3	1520 ± 55	1509 ± 35	279 ± 19	290 ± 18	115 ± 12	121 ± 12
SpillOver	450 ± 21	980* ± 72	861 ± 74	213* ± 12	193 ± 13	2113 ± 119	2131 ± 128	395 ± 36	400 ± 42	182 ± 14	182 ± 33

Summary of the average body mass along with the average muscle wet mass of the tibialis anterior (TA), extensor digitorum longus (EDL), gastrocnemius (GAST), plantaris (PLA) and soleus (SOL) muscles. Data is given for a group of untrained animals (n = 5), a group of animals trained with unloaded concentric contractions (UNL, n = 5) and a group trained with antagonistic co-contraction (SpillOver, n = 5). The training of the animals was always conducted on the left side. Data is presented as mean ± SD.

Muscles with a statistically (p < 0.05) higher mass than the respective control muscles are indicated with an asterisk (*).

In the UNL trained group, there was no significant difference between the mean mass of the stimulated TA and EDL and the unstimulated control side although the means increased in the expected direction (TA by 5.4 ± 2.5%, p = 0.199 and EDL by 3.6 ± 1.3%, p = 0.126). The SpillOver stimulated group on the other hand revealed significantly higher muscle wet mass for the stimulated TA (13.9 ± 1.3%, p = 0.034) and EDL (10.7 ± 1.8%, p = 0.029) muscles. Data presented as mean relative increase of muscle wet mass ± SEM.

**Fig 7:**
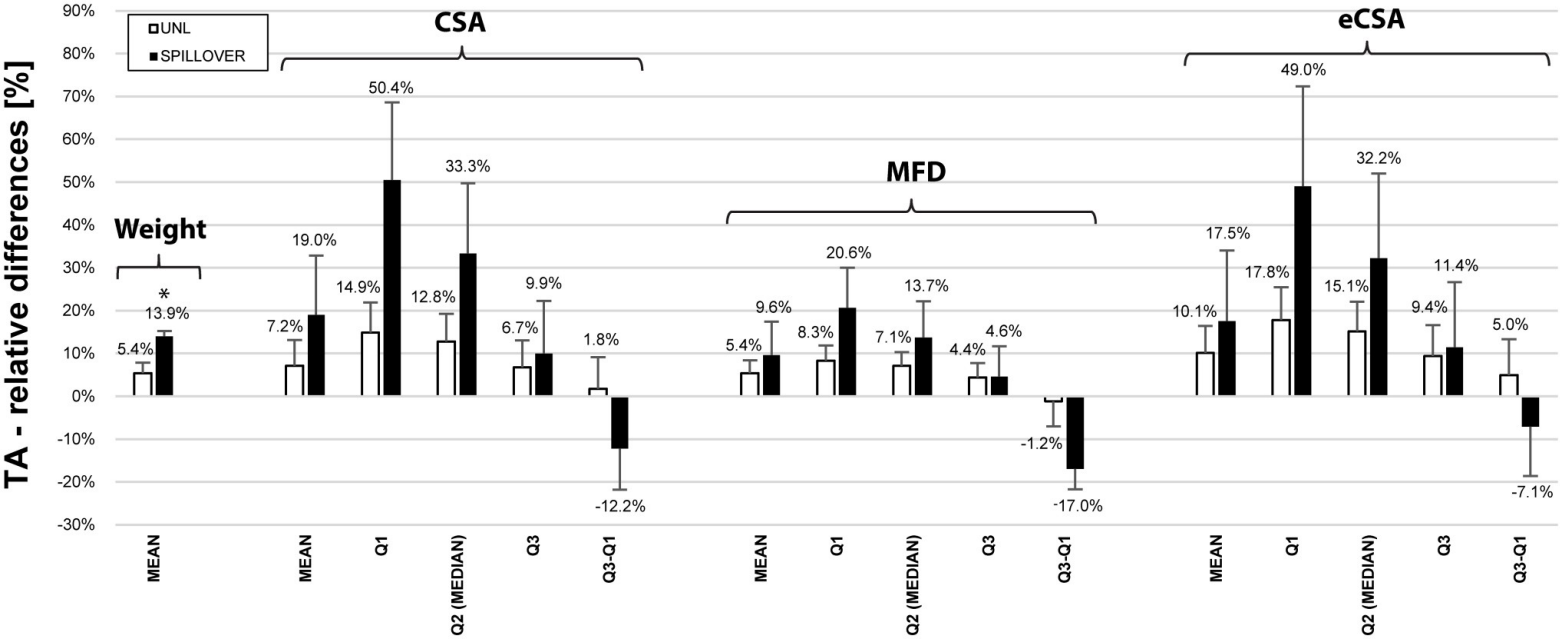
Relative differences in mass and muscle fibre morphology of the tibialis anterior muscle. Relative differences between stimulated and control side of the tibialis anterior muscle for animals chronically trained with either unloaded concentric contractions (UNL, white bars, n = 5) or antagonistic co-contractions (SpillOver, black bars, n = 5). Fiber cross sectional area (CSA), minimal Feret’s diameter (MFD) and an estimated fiber cross sectional area (eCSA) were obtained for each cell of each muscle. Histologic results were compared regarding mean, first quartile (Q1), median (Q2), third quartile (Q3) and inter-quartile-distance (Q1-Q3). Data is presented as mean relative difference ± SEM with positive differences representing a greater value in the stimulated muscles. Statistical significance (p < 0.05) is indicated by an asterisk (*) symbol.

**Fig 8:**
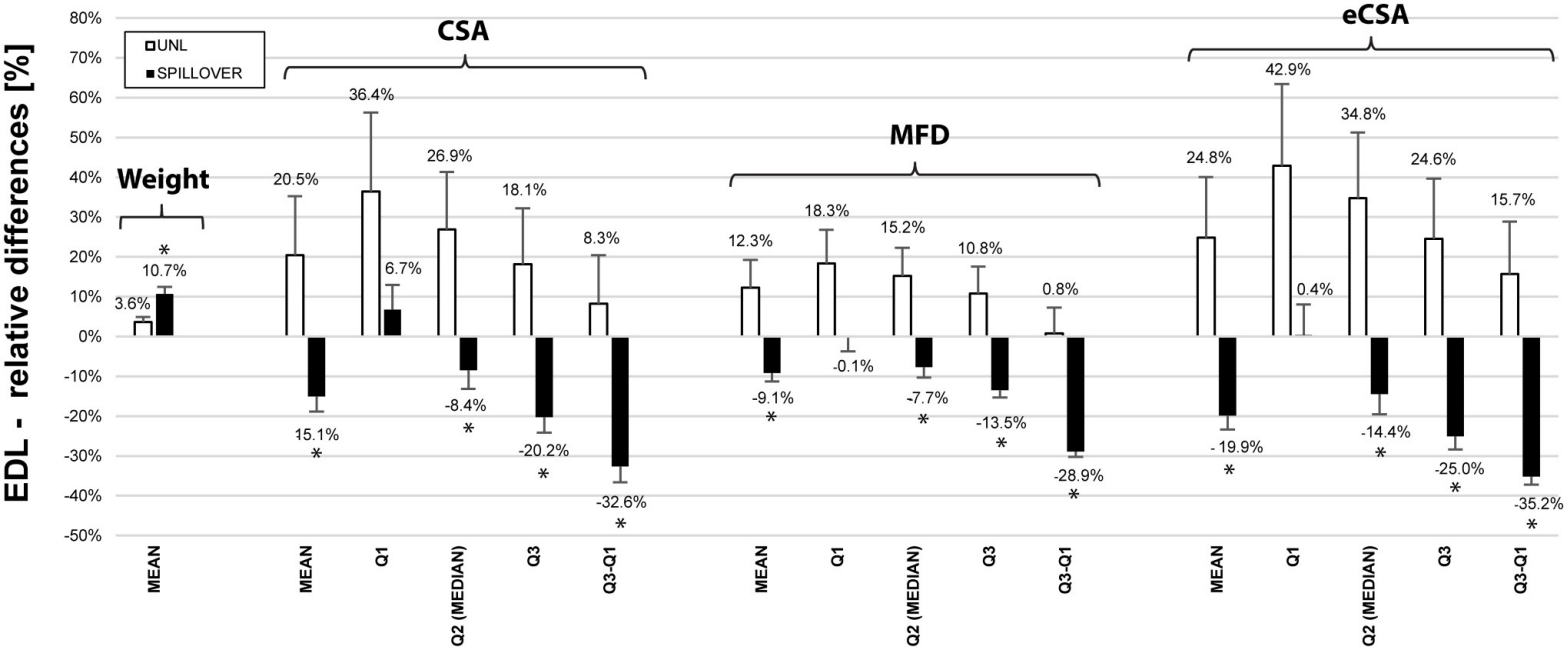
Relative differences in mass and muscle fibre morphology of the extensor digitorum longus muscle. Relative differences between stimulated and control side of the extensor digitorum longus muscle for animals chronically trained with either unloaded concentric contractions (UNL, white bars, n = 5) or antagonistic co-contractions (SpillOver, black bars, n = 5). Fiber cross sectional area (CSA), minimal Feret’s diameter (MFD) and an estimated fiber cross sectional area (eCSA) were obtained for each cell of each muscle. Histologic results were compared regarding mean, first quartile (Q1), median (Q2), third quartile (Q3) and inter-quartile-distance (Q1-Q3). Data is presented as mean relative difference ± SEM with positive differences representing a greater value in the stimulated muscles. Statistical significance (p < 0.05) is indicated by an asterisk (*) symbol.

SpillOver training showed significantly higher increases in wet muscle mass than in UNL trained animals for the TA (p = 0.015) and EDL (p = 0.013) muscle (see Figs 7 and 8). No significant changes in muscle mass were seen in the plantar-flexor muscles GAST (p = 0.818), PLA (p = 0.864) or SOL (p = 0.535).

#### Histology

Summaries of the histologic analysis were expressed as average relative differences ± SEM between trained and untrained side for the TA muscle (see Fig 7) and the EDL muscle (see Fig 8). Histograms of CSA, MFD and eCSA can be found as supporting material for each individual animal in S1 Histograms.

For the TA muscle, analysis of mean CSA, MFD and eCSA showed a clear trend for greater increases in the SpillOver stimulated group than in the UNL stimulated group, but didnʹt reach statistical significance (p_CSA_ = 0.455, p_MFD_ = 0.630, p_eCSA_ = 0.684). Generally higher gains were observed for first and second quartile, suggesting a potentially stronger growth of small muscle fibers. Differences were less pronounced for the third quartile. SpillOver stimulation additionally showed a narrowing in the spread of data for all parameters, as indicated by a decrease of the inter-quartile distance. UNL contractions had no influence on the distribution of data.

The histological differences between the UNL and the SpillOver trained group showed a clear trend towards greater cellular hypertrophy using SpillOver stimulation but unlike the data for muscle wet mass did not reach statistical significance for any of the observed parameters.

For the EDL muscle, the relative differences were contrary between the UNL and SpillOver group. While CSA, MFD and eCSA of the fibers in the UNL group generally were increased in size, they decreased in the SpillOver group. In this case, the differences between UNL and SpillOver group were significant (p < 0.05), except the results for the first quartile (p_CSA_ = 0.192, p_MFD_ = 0.081, p_eCSA_ = 0.089). In the UNL group the first quartile showed the highest gains, suggesting a higher impact on the smaller diameter fibers. While in contrast the SpillOver stimulated group showed only little changes in the first quartile, very distinct decreases could be observed in the third quartile. An effect, which was already seen in analysis of the TA muscle, was the narrowing of the distributions in the SpillOver group as indicated by the inter-quartile distance. For the EDL muscle this effect was even more pronounced. The changes in muscle fiber cross sectional area are thus not simply correlated with the significant difference in muscle wet mass.

## Discussion

We demonstrated a novel technique to elicit resisted or loaded contractions of the dorsiflexor muscles in the rat hind limb, by means of a single channel electrical nerve stimulator. The amount of load acting on the TA muscle was investigated in a series of acute experiments under anesthesia, comparing tensile forces measured during unloaded concentric contractions, isometric contractions and antagonistic co-contractions.

Charge-balanced monophasic pulses were used to elicit single twitches to acquire recruitment curves for the different loading regimes. Both isometric and SpillOver contractions produced significantly higher twitch-peak-forces in the TA than in the unloaded case. Interestingly SpillOver twitches were not able to reach ISO peak-forces. Although the much stronger plantar-flexor muscles were working against the fully recruited TA muscle it seemed that the 20 ms duration of a single twitch contraction of the relatively fast dorsiflexors was not sufficient to allow a resisting force to develop completely. The plantarflexors are slower to generate force than the dorsiflexors.

Normalizing the maximal tetanic forces to the wet mass of the muscle gives 8.7 ± 1.1 N g^-1^ for unloaded, 16.9 ± 2.7 N g^-1^ for isometric and 20.8 ± 2.7 N g^-1^ for SpillOver contractions (data presented as mean ± SD). Our results for isometric contractions are in general agreement with results reported for isometric contractions in other studies, e.g. 15.9 N g^-1^ [30] (SD not reported), 15.1 ± 1.6–16.3 ± 1.0 N g^-1^ [31] and 17.2 N g^-1^ [32] (SD not reported), for adult rats using transducers fixed to an external mechanical frame. These transducers require a substantial rearrangement of the biomechanical system which eliminates the possibility to measure forces generated during unloaded concentric contractions or co-contractions. Comparable results for the other tested modalities (UNL, SpillOver) are not available in the literature–our data is novel. However the congruence between our isometric values and previously published values gives us confidence that the values measured in the UNL and SpillOver cases are reliable.

Using a single frequency ramped burst offers a time-saving possibility to assess the force-frequency relationship of a particular muscle. The measured peak-forces within each frequency segment of the burst were slightly lower compared to the standard approach. This can be explained by the shorter stimulation durations for each frequency level, which gave the muscle less time to generate force. The shorter durations were necessary to minimize fatigue within the burst. Nevertheless this alternative approach provides a quick estimate of the force-frequency relationship which is comparable to the standard-approach, reducing the measurement time from minutes to seconds.

To investigate the effect of loading on the hypertrophic response, the TA muscle was artificially activated by means of functional electrical nerve stimulation. The animals received a high intensity training pattern challenging the TA muscle by performing either unloaded contractions (CPN stimulation) or antagonistic co-contractions (SpillOver stimulation). The selected stimulation amplitude of the SpillOver group was able to produce forceful contractions, which were initially concentric or isometric for the TA muscle, i.e. the ankle angle changed little during stimulation, or produced a slight dorsiflexion. With increasing fatigue, the influence of the much stronger plantar-flexor muscles became more dominant resulting in an eccentric movement, i.e. a plantarflexion during stimulation. Contractions of the UNL group were exclusively concentric.

The SpillOver trained group showed significant increases in muscular wet mass of the TA (13.9 ± 2.9%, mean ± SD) and EDL (10.7 ± 4%, mean ± SD) muscles. There were no differences in mass for plantar-flexor muscles (GAST, PLA, SOL). We assume that the additional activity caused by stimulation of only some of the motor units in the plantar flexor muscles was not sufficient to elicit hypertrophy in those muscles.

In 1988, Wong and Booth developed an external apparatus to train the electrically activated hind limb muscles of a rats which acted against a weighted plate [23]. Although their main focus was to generate hypertrophy in the planta-flexor muscles, they also achieved a significant increase in mass of the TA by 16% compared to the contralateral control-side. In an additional group which received the same stimulation protocol but was not working against an extra load, the muscle mass of the plantar-flexors was not affected, while the mass of the TA muscle again increased by 17%. They observed that the electrical stimulations caused a certain degree of co-contraction which could have caused additional of loading of the TA muscle explaining the observed hypertrophy. In a subsequent study 1990, they used their setup to study the differences between unloaded and high-resistance concentric contractions of the gastrocnemius muscle in a high-frequency (192 repetitions) training protocol [24]. They reported an increase in muscle wet mass of the stimulated TA of 16% for the unloaded group and 30% for the high-resistance group, in comparison to a sedentary control group. These values need to be interpreted with care, as also the unstimulated muscles of the trained animals showed a significant growth. Comparing the average muscle mass of stimulated versus not stimulated side within each group, gives a relative increase of 6% for the unloaded group and 13% for the high resistance group (values calculated on basis of the reported average muscle mass and therefore consistent with the results from training in the awake rat reported here). 1990 Baar and Esser used a setup in which they enforced resistance training by stimulation of the sciatic nerve to elicit a simultaneous contraction of all innervated hind limb muscles twice a week for a duration of 6 weeks [22]. They reported a significant increase of muscle mass of the TA by 14.4 ± 3.15% and EDL by 13.9 ± 3.01%. Our results correlate well with the results currently published in literature, demonstrating that our model is suitable to generate an appropriate stimulus to trigger hypertrophy, but without the need for repeated anesthesia.

Limitations of the present study were differences in age and weight between the UNTRAINED, UNL and SpillOver group. To compensate for these inter-individual differences we only evaluated relative changes between left (trained) and right (control) leg of the same animal. Further we normalized muscle mass of the control TA and EDL muscles to the animalʹs body mass. A one-way ANOVA did not detect a statistical difference in the normalized muscle mass of the evaluated the TA (p = 0.507) or EDL (p = 0.874) control muscles for any of the groups. Although we cannot exclude a potential influence of the physiological group differences, we assume that our conclusions are reliable based on the similar normalized masses of the observed muscles.

Differences in CSA, MFD and eCSA of the TA muscle were generally more pronounced in the SpillOver stimulated group. The biggest changes were observed for the first and second quartile, suggesting that TA muscle fibers with a small diameter were more affected by the training. The SpillOver training revealed decreased inter-quartile distances for all parameters resulting in narrower distributions which can be explained by a stronger shift towards bigger fiber size in the lower quartiles. This effect was not the seen in the UNL group.

The histologic analysis of the TA muscles was not able to detect significant differences between the UNL and SpillOver group, but it shows a clear trend illustrating that a higher degree of loading is advantageous for muscular enlargement. Visual inspection of the histologic sections did not show any signs of muscle damage (no central nuclei or increased amount of connective tissue) or edema in the stimulated muscle.

For the extensor digitorum longus muscles, the analysis revealed unexpected differences between the UNL and the SpillOver group. Similar to the TA muscles, the EDL muscles of the SpillOver stimulated group showed a significantly higher muscle wet mass (p = 0.013) compared to the UNL stimulated group. Interestingly these greater gains could not be attributed to increases in muscle fiber diameter, at least in the mid belly cross sections that we analyzed. While in the UNL group the muscle fibers showed a clear increase in all observed parameters, the opposite (decrease in CSA and MFD) was the case for the SpillOver group.

It can be argued that the EDL muscle experiences a higher level of stretch during SpillOver stimulation as it is acting over two joints (knee and ankle). During UNL and SpillOver induced contractions the knee is fully flexed which is stretching the EDL via its proximal tendon. Additional stretch from the distal tendon was experienced during plantarflexion of the ankle which was only present in the SpillOver group.

Visual inspection of the muscle sections (H&E stained) showed no signs of acute inflammation, swelling, muscle damage (no central nuclei) nor an increased amount of connective tissue compared to the control side. The muscle cells on the SpillOver stimulated side appeared to be smaller in size and generally rounder in shape.

In the past, it has been shown that eccentric contractions can cause a shift of the muscleʹs optimal length by increasing the number of sarcomeres within a muscle fiber [33–36], which might provide a partial explanation for the higher gains in wet mass of EDL muscles without increasing the fiber diameter. Another interpretation of the increased number of small muscle fibers observed in the EDL muscles of the SpillOver group could be the *de novo* formation of muscle fibers. Antionio and Gonyea reviewed the literature with respect to models using compensatory hypertrophy, stretch or exercise to induce muscular growth [37], in which they found increases in fiber number in the range of 9–82%. Considering only studies performing weight lifting exercises on rats, elevated fiber counts were reported between 12–14% [13,15]. However, in the present study we did not quantify the number of sarcomeres or the number of muscle fibers. Additional analysis in future experiments could provide valuable information about the actual mechanisms involved.

The main advantage using SpillOver stimulation is that the loading of the dorsi-flexor muscles can be controlled over a wide range by only changing the electrical stimulation parameters. This novel technique can elicit concentric, isometric and eccentric contractions which was illustrated in a series of acute force measurements. In combination with modern remotely programmable IPGʹs, SpillOver stimulation can be used to realize protocols for resistance training in the freely moving rat. Such a training can be delivered automatically resulting in less stress for the animal, compared to the repetitive anesthesia and manual interventions performed in previous studies [22–24]. The training was tolerated very well by all animals, they did not show abnormal social behavior or signs of distress in response to the stimulation.

In the present study the stimulation amplitude was constant throughout the entire duration of the experiment. We hypothesize that even higher gains in hypertrophy may be possible by adapting the stimulation pattern and intensity over time–implementing the principles of progressive resistance training.

## Conclusion

We report a novel model to generate hypertrophy in the rat TA and EDL muscle using SpillOver stimulation to elicit adjustable co-contractions of the dorsiflexor and plantarflexor muscles. The amount of load acting on the TA muscle was assessed in acute experiments, measuring tensile forces in a series of short bursts of varying stimulation frequency and amplitude. This technique was able to generate concentric, isometric and eccentric contractions within a single setup.

The effect of loading on the hypertrophic response was tested in animals which received a high-intensity daily training pattern, challenging the TA muscle by performing either unloaded concentric contractions or antagonistic co-contractions. Both groups showed an increased muscle wet mass of the TA muscle with significantly higher gains in the SpillOver group. Our results correlate well with the results currently published in literature, demonstrating that our model is suitable to trigger hypertrophy. We hypothesize that even higher gains in muscle mass may be possible by optimizing the stimulation parameters according to the principles of progressive resistance training.

## Supporting information

S1 Table Physiological force measurements. Overview of twitch and tetanic peak forces measured during unloaded concentric (UNL), isometric or antagonistic co-contractions (SpillOver).
(PDF)

S2 Table Conditioning pattern—Normalized peak forces and decline. Overview of peak-forces, normalized to the peak-force of the first contraction, measured for a conditioning pattern delivered using SpillOver Stimulation at 1 mA and 100 Hz. The pattern consisted of 5 sets with 10 repetitions (2 s contraction followed by 2 s rest) and 2.5 min rest between the sets. This pattern was used as training pattern in the hypertrophy experiments.
(PDF)

S1 Histograms Histologic analysis. Overview for fiber cross sectional area (CSA), minimal Feret’s diameter (MFD) and estimated fiber cross sectional area (eCSA) of the tibialis anterior muscle and the extensor digitorum longus muscle. Histograms are given for the unloaded (UNL) and SpillOver training regime comparing untrained and trained side.
(PDF)
