# Supplementary material for: SpillOver stimulation: A novel hypertrophy model using co-contraction of the plantar-flexors to load the tibial anterior muscle in rats
Source: PLoS One. 2018 Nov 20;13(11):e0207886. doi: 10.1371/journal.pone.0207886 (PMC6245836; doi:10.1371/journal.pone.0207886)
Supplement: S1 Table — Overview of twitch and tetanic peak forces measured during unloaded concentric (UNL), isometric or antagonistic co-contractions (SpillOver). (PDF) [file pone.0207886.s001.pdf]

Animal	F_{peak} twitch UNL	F_{peak} twitch ISO	F_{peak} twitch SpillOver	F_{peak} tetanic UNL	F_{peak} tetanic ISO	F_{peak} tetanic SpillOver
#	N	N	N	N	N	N
1	0.79	2.41	1.25	4.05	7.85	11.11
2	0.89	1.64	1.05	5.77	10.64	10.21
3	0.96	2.63	1.71	5.66	9.87	13.91
4	0.85	2.91	1.38	5.42	11.87	14.18
5	1.30	3.22	1.88	6.25	12.18	15.36
Mean	0.96	2.56	1.45	5.43	10.48	12.95
SD	0.20	0.60	0.34	0.83	1.74	2.19

Physiological force measurements Overview of twitch and tetanic peak forces measured during unloaded concentric (UNL), isometric or antagonistic co-contractions (SpillOver).
