# Supplementary material for: SpillOver stimulation: A novel hypertrophy model using co-contraction of the plantar-flexors to load the tibial anterior muscle in rats
Source: PLoS One. 2018 Nov 20;13(11):e0207886. doi: 10.1371/journal.pone.0207886 (PMC6245836; doi:10.1371/journal.pone.0207886)
Supplement: S2 Table — Overview of peak-forces, normalized to the peak-force of the first contraction, measured for a conditioning pattern delivered using SpillOver Stimulation at 1 mA and 100 Hz. The pattern consisted of 5 sets with 10 repetitions (2 s contraction followed by 2 s rest) and 2.5 min rest between the sets. This pattern was used as training pattern in the hypertrophy experiments. (PDF) [file pone.0207886.s002.pdf]

	Maximal F_{peak}					Decline of F_{peak} within the set				
Animal	Set 1	Set 2	Set 3	Set 4	Set 5	Set 1	Set 2	Set 3	Set 4	Set 5
#	%	%	%	%	%	%	%	%	%	%
1	100.0	91.8	81.9	81.9	80.6	41.4	41.9	36.9	37.1	41.7
2	100.0	96.0	87.3	82.2	81.4	48.8	49.6	45.0	40.3	40.3
3	100.0	83.9	81.1	82.2	81.4	47.1	37.3	35.2	35.6	36.3
4	100.0	84.2	78.8	73.7	72.6	39.9	39.1	39.5	38.1	38.1
5	100.0	75.7	61.5	54.1	57.2	75.9	60.0	51.3	43.9	48.1
Mean	100.0	86.3	78.1	74.8	74.6	50.6	45.6	41.6	39.0	40.9
SD	0.0	7.9	9.8	12.1	10.4	14.6	9.4	6.6	3.2	4.5

Conditioning pattern - normalized peak forces and decline Overview of peak-forces, normalized to the peak-force of the first contraction, measured for a conditioning pattern delivered using SpillOver Stimulation at 1 mA and 100 Hz. The pattern consisted of 5 sets with 10 repetitions (2 s contraction followed by 2 s rest) and 2.5 min rest between the sets. This pattern was used as training pattern in the hypertrophy experiments.
