# Supplementary material for: SpillOver stimulation: A novel hypertrophy model using co-contraction of the plantar-flexors to load the tibial anterior muscle in rats
Source: PLoS One. 2018 Nov 20;13(11):e0207886. doi: 10.1371/journal.pone.0207886 (PMC6245836; doi:10.1371/journal.pone.0207886)
Supplement: S1 Histograms — Overview for fiber cross sectional area (CSA), minimal Feret’s diameter (MFD) and estimated fiber cross sectional area (eCSA) of the tibialis anterior muscle and the extensor digitorum longus muscle. Histograms are given for the unloaded (UNL) and SpillOver training regime comparing untrained and trained side. (PDF) [file pone.0207886.s003.pdf]

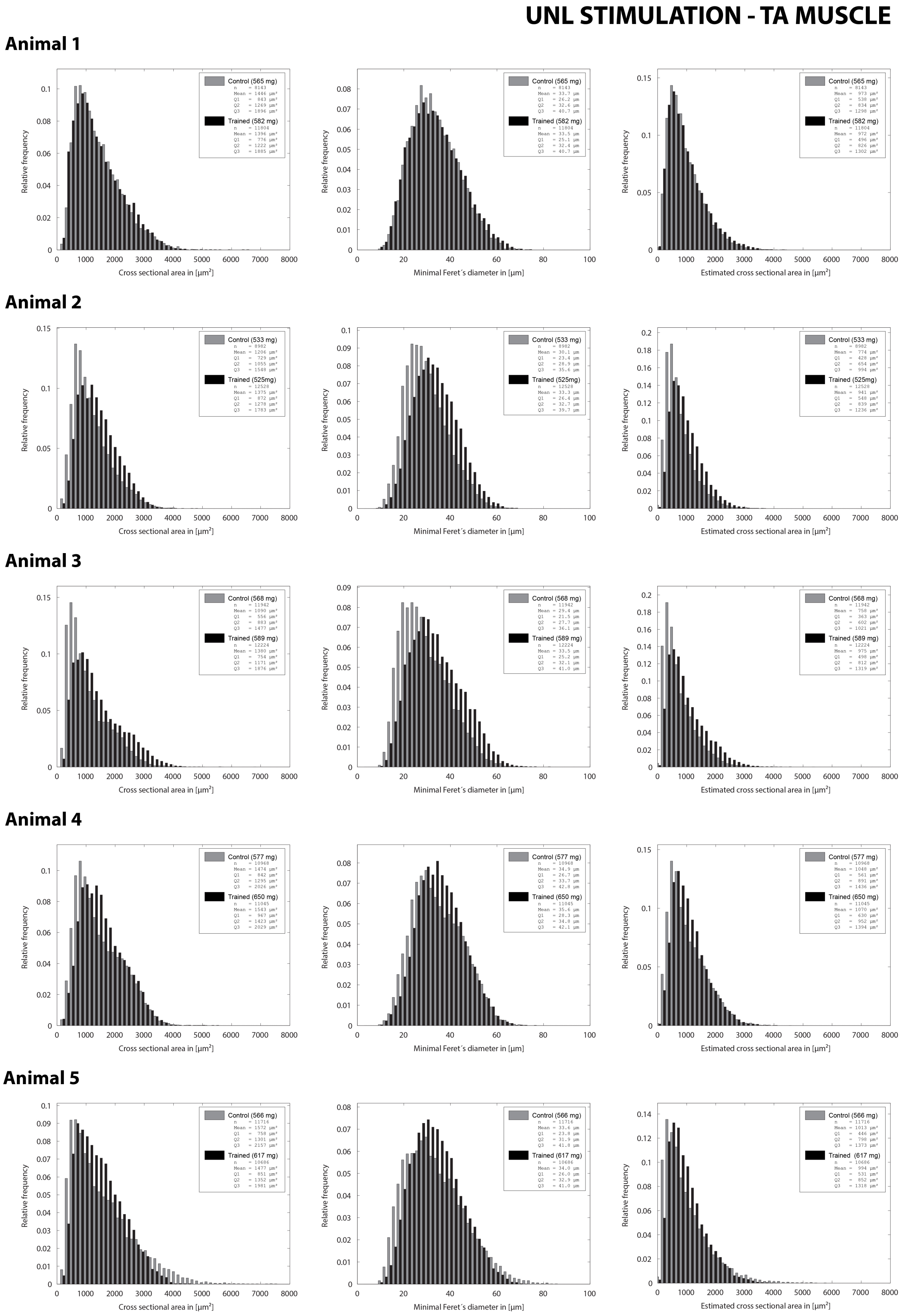

Illustration 1. Histologic analysis of the TA muscle - UNL group Overview for fiber cross sectional area (CSA), minimal Feret's diameter (MFD) and estimated fiber cross sectional area (eCSA) of the tibialis anterior muscle. Histograms for untrained (grey) and trained (black) side were superimposed for direct comparison.

UNL STIMULATION - EDL

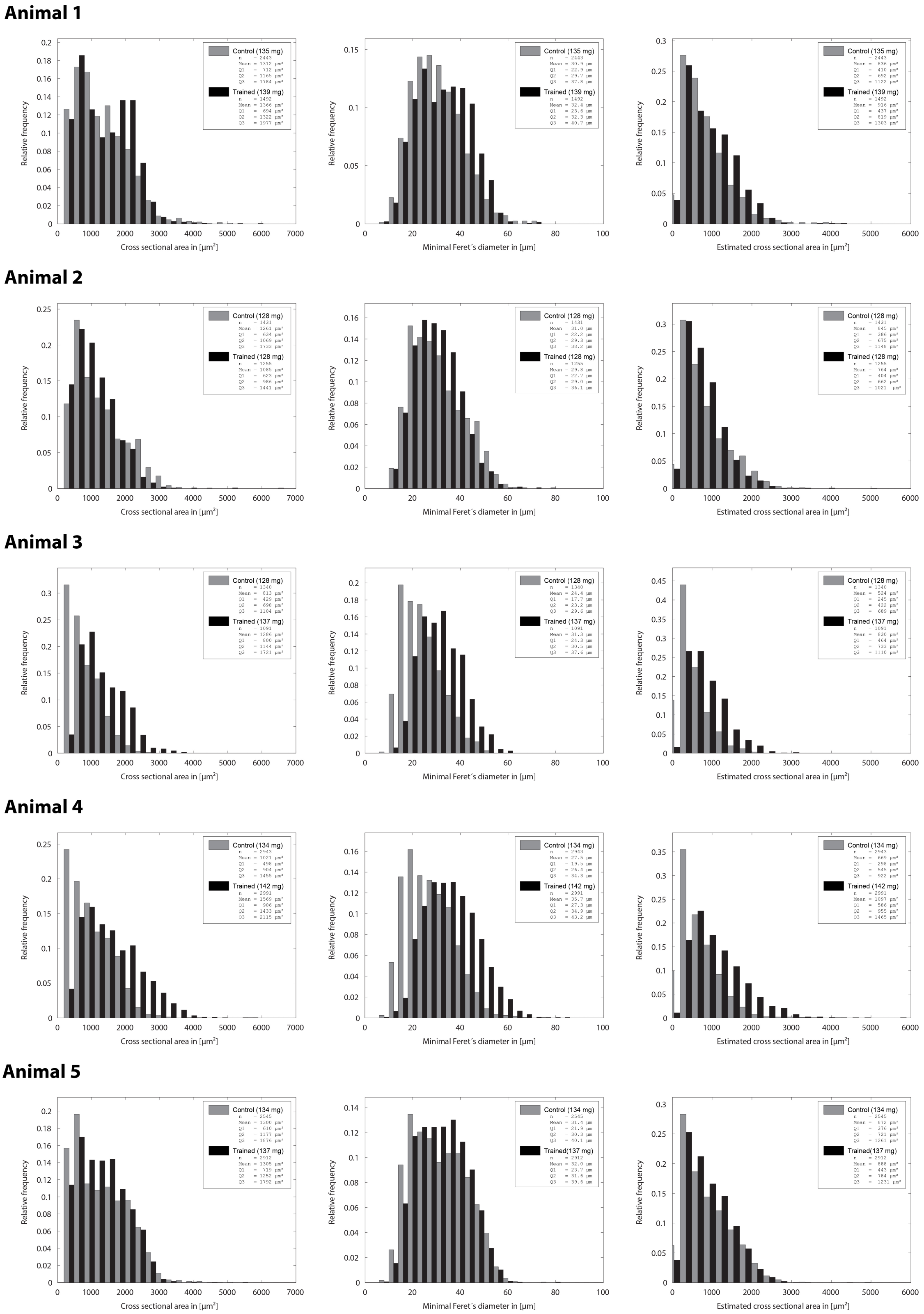

Illustration 2. Histologic analysis of the EDL muscle - UNL group Overview for fiber cross sectional area (CSA), minimal Feret’s diameter (MFD) and estimated fiber cross sectional area (eCSA) of the tibialis anterior muscle. Histograms for untrained (grey) and trained (black) side were superimposed for direct comparison.

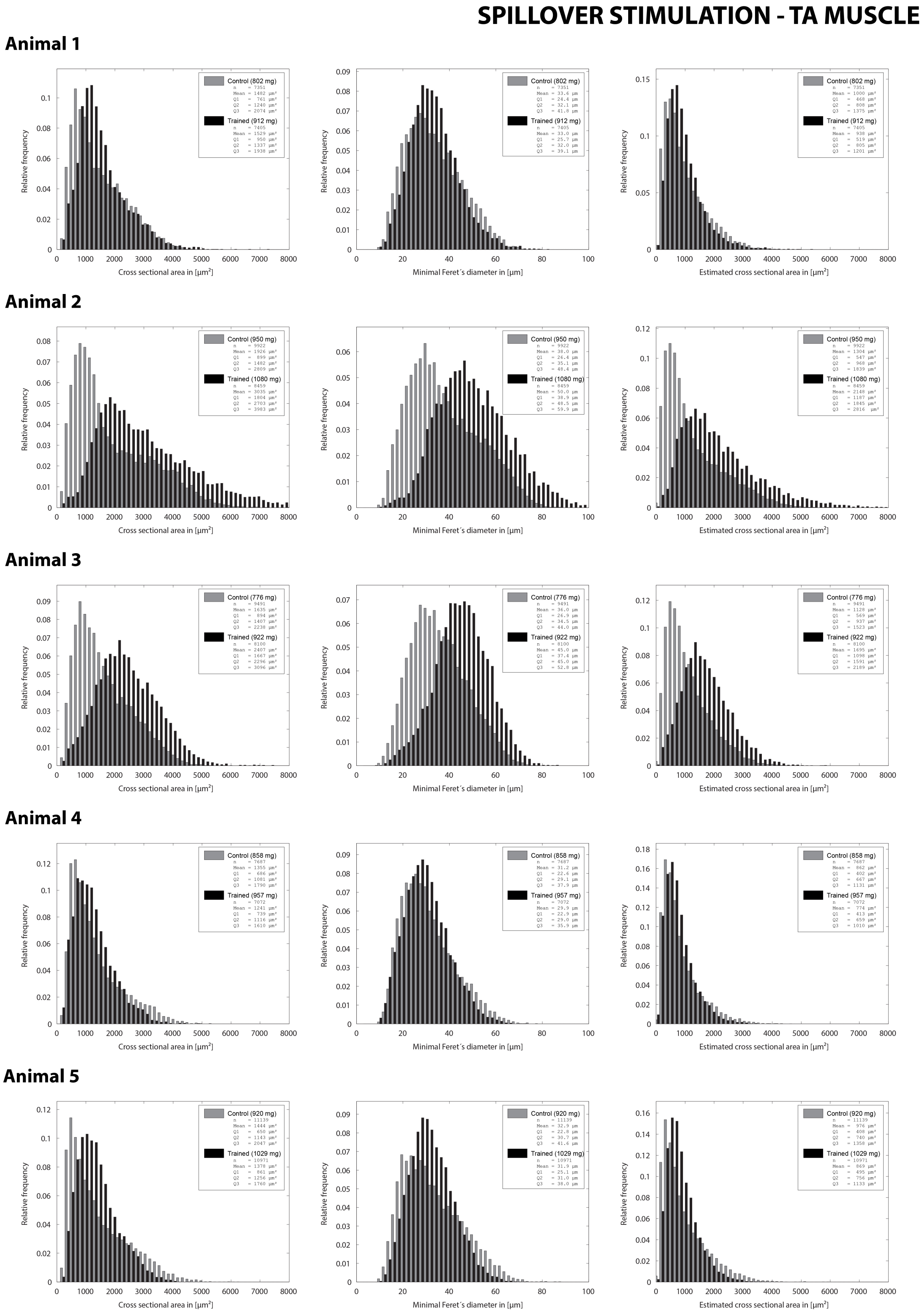

Illustration 3. Histologic analysis of the TA muscle - SpillOver group Overview for fiber cross sectional area (CSA), minimal Feret's diameter (MFD) and estimated fiber cross sectional area (eCSA) of the tibialis anterior muscle. Histograms for untrained (grey) and trained (black) side were superimposed for direct comparison.

SPILLOVER STIMULATION - EDL

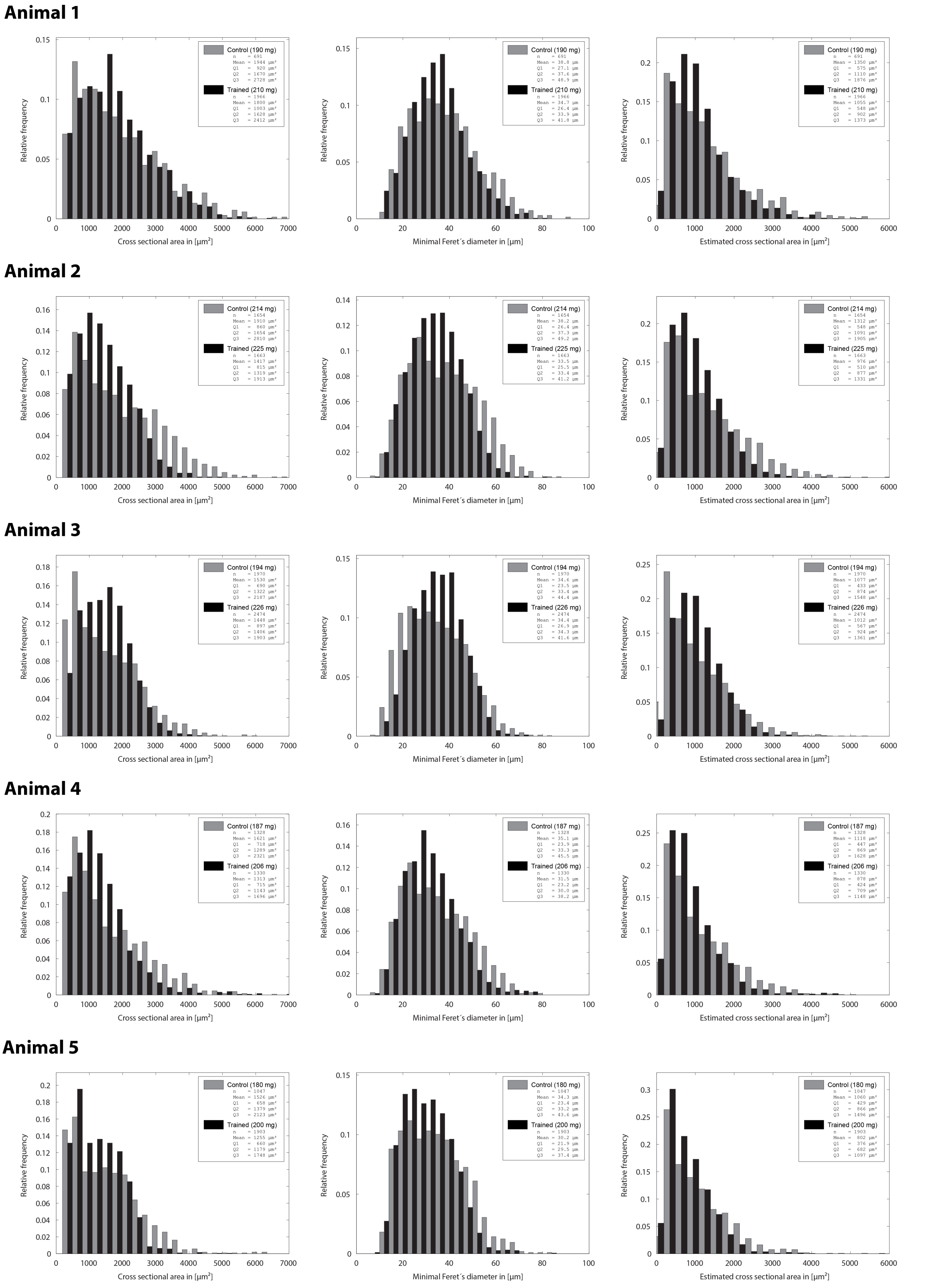

Illustration 4. Histologic analysis of the EDL muscle - SpillOver group Overview for fiber cross sectional area (CSA), minimal Feret’s diameter (MFD) and estimated fiber cross sectional area (eCSA) of the tibialis anterior muscle. Histograms for untrained (grey) and trained (black) side were superimposed for direct comparison.
